# Age-Related Expression of the Polymeric Immunoglobulin Receptor (pIgR) in the Gastric Mucosa of Young Pigs

**DOI:** 10.1371/journal.pone.0081473

**Published:** 2013-11-13

**Authors:** Paolo Trevisi, Greta Gandolfi, Davide Priori, Stefano Messori, Michela Colombo, Maurizio Mazzoni, Jean-Paul Lallès, Paolo Bosi

**Affiliations:** 1 Department of Agricultural and Food Sciences, University of Bologna, Reggio Emilia, Italy; 2 Department of Veterinary Medical Science, University of Bologna, Ozzano dell’Emilia, Italy; 3 Food and Digestive, Central and Behavioral Adaptation Department, French National Institute for Research in Agriculture, Saint-Gilles, France; University of Houston, United States of America

## Abstract

To date few studies have addressed the development and function of the porcine gastric mucosal immune system and this is a major limitation to understanding the immunopathogenesis of infections occurring in young pigs. The polymeric immunoglobulin receptor (pIgR) mediates the transport of secretory immunoglobulins until luminal surface of the gut mucosa and the aim of this study was to investigate the time course of pIgR expression and to determine its localization in three functionally different porcine gastric sites during the suckling period and after weaning. An additional goal was to investigate the time course expression of toll-like receptors (TLRs) in relation to pIgR expression. Gastric samples were collected from the cardiac-to-oxyntic transition (Cd), the oxyntic (Ox), and the pyloric (Py) regions in 84 pigs, slaughtered before weaning (14, 21 and 28 days of age; 23, 23 and 19 pigs, respectively) and 14 days post-weaning (42 days of age, 23 pigs). *PIgR* was expressed in the mucosa of all the three gastric sites, and its transcript levels were modulated during suckling and after weaning, with regional differences. *PIgR* expression increased linearly during suckling (*P*=0.019) and also increased post-weaning (*P*=0.001) in Cd, it increased post-weaning in Py (*P*=0.049) and increased linearly during suckling in Ox (*P*=0.036). TLRs expression was also modulated during development: in Cd, *TLR2* increased linearly during suckling (P=0.003); in Ox, *TLR2* decreased after weaning (P=0.038) while *TLR4* increased linearly during suckling(P=0.008). The expression of *TLR2*, 3 and 4 in Ox was positively correlated with *pIgR* expression (P<0.001).

Importantly, both pIgR protein and mRNA were localized, by immunohistochemistry and in situ hybridization, respectively, in the gastric glands of the lamina propria. These results indicate that pIgR is actively synthesized in the gastric mucosa and suggest that pIgR could play a crucial role in gastric mucosal immune defense of growing pigs.

## Introduction

The mucosal immune system of the gastrointestinal tract plays a primary role in the interaction between the host and both commensal and potentially pathogenic microorganisms [1]. After birth and at weaning, the digestive system of pigs is exposed to novel antigens and the appropriate development and tuning of immune responses is critical to provide an active response against infections and to maintain tolerance to harmless antigens and commensal bacteria [2]. Inadequate immune responses to pathogens or inappropriate active reactions against harmless antigens can impair piglet’s development and its production performances [3].

A key element of the adaptive mucosal immune system is the polymeric immunoglobulins (pIgs). They are produced by plasma cells in the lamina propria and are actively transcytosed to the lumen through the epithelial cell by the polymeric immunoglobulin receptor (pIgR) [4]. The pIgR is a transmembrane glycoprotein synthesized by epithelial cells lining mucous membranes and exocrine glands. The pIgR binds polymeric immunoglobulin A (pIgA), and to a lesser extent immunoglobulin M (pIgM) at the basolateral surface of cells. The pIgR mediates pIg transcytosis to the apical surface where the pIgs-pIgR complex is cleaved to generate secretory immunoglobulins (sIgs). Consequently, sIgs released into the lumen are composed of the pIgs and the extracellular portion of pIgR, defined as secretory component or SC [4,5]. In the absence of specific immunoglobulin production, SC is released in its free form into the lumen, representing an important component of the innate anti-microbial defense [6]. The pIgR is critical for the maintenance of mucosal homeostasis and food tolerance in mice [7] and high levels of expression have been observed in the intestine with low levels in the lung, kidney, pancreas and endometrium in humans [8], and in liver and stomach of mice [9]. To date, mucosal immune responses in the stomach have been largely neglected, often as they were considered to be of minor importance in relation to gut diseases, due to the inhospitable microbial environment and to the shorter exposure time to the feed, in comparison with other regions of the gastrointestinal tract. There are no previous reports on the expression of pIgR in pigs, however, the occurrence and distribution of lymphoid follicles in functionally different gastric sites in piglets suggested the existence of the basic machinery for adaptive immune response in the stomach [10]. Since the stomach is the main portal to the intestine and can be reasonably considered as the first line of defense we chose to investigate the role of the porcine stomach in the defense against pathogens. To the best of our knowledge, no previous studies have characterized the expression of pIgR during gastric mucosa development in the suckling period and after weaning in pigs.

The induction of pIgR expression is dependent upon multiple mechanisms at both transcriptional and post-transcriptional levels [11]. A major determinant is likely to involve the detection of microbiota in the gut lumen by several pattern recognition receptors, including Toll-like receptors (TLRs). Thus, the presence and the progressive modulation of TLRs activity during gastric colonization by enteric bacteria may be critical for the regulation of pIgR expression and for the general development of a gastric-associated immune system. Therefore, the present study aimed to: a) determine whether pIgR was expressed in the stomach of piglets during the suckling period and after weaning; b) investigate the time course pIgR mRNA expression in three functionally different sites; c) analyze the time course of mRNA concurrent expression of TLRs in these sites; d) investigate pIgR protein distribution in these gastric sites; and finally, e) confirm the active synthesis of pIgR in the gastric mucosa by mRNA localization at sites of pIgR protein staining.

## Materials and Methods

### Ethics statement

The experimental protocol was designed in compliance with recommendations of the French law (Decree: 2001-464 29/05/01) and of the European Community (Directive 86/609/EEC) for the care and use of laboratory animals. The authorization n° 006708 from the French Veterinary Services is the personal agreement of one of the co-authors (JPL). It covers all aspects of experimentation carried out by this researcher, including zootechnical trials as well as surgery, any substance administration and sampling of biological fluids on live farm animals (ruminants and monogastrics, including pigs), euthanasia and collection of samples of biological fluids and tissues after slaughter. The authorization (n° A35-622) as issued by the French Veterinary Services is attributed to the experimental facilities of the Institut National de la Recherche Agronomique (INRA) located in Saint-Gilles, France, where the *in vivo* experiment and the slaughter of pigs was carried out. The sows were not sacrificed as part of the present study.

### Experimental design, pig slaughter and tissue sampling

In this trial, 84 piglets obtained from 23 Large White x Landrace sows, inseminated with Piétrain semen were recruited. Eleven sows were treated orally with the antibiotic amoxicillin from day -10 pre-farrowing to day 21 post-farrowing, while twelve sows were untreated. The piglets were randomly slaughtered, balanced per sow and treatment, at 14, 21 and 28 days of age (23, 23 and 19 pigs, respectively), or at 42 days of age (14 days post-weaning; 19 pigs). For the post-weaning groups, litters were kept separate from each other and piglets received a standard weaning diet without addition of antibiotics or growth promoters.

At slaughter, the piglets were stunned by electric shock and killed by exsanguination 1 h after the last meal. For each pig, a midline abdominal incision was made and the stomach was gently removed. The stomach was opened along the greater curvature, emptied of its contents, and rinsed with double-distilled water.

Tissue samples were collected in three functionally different sites of the stomach: i. the cardiac-to-oxyntic mucosa transition region in the lesser curvature, ii. the oxyntic mucosa (the proper gastric gland region in the body) and iii. the pyloric mucosa in the antrum. These three gastric sites are referred to as Cd, Ox, and Py, respectively, in the present report.

For RNA extraction and expression analysis, samples from these gastric sites were collected, immediately snap frozen in liquid nitrogen and stored at -80 °C until analysis. For immunohistochemistry and *in situ* hybridization analyses, whole thickness tissue specimens of ≈1 cm^2^ were pinned tightly to balsa wood, fixed in 10% buffered formalin for 24 h, dehydrated in a graded series of ethanol and finally embedded in paraffin wax.

### Real-time quantitative PCR

Total RNA was isolated from tissue samples according to the Takara Fast Pure kit (Takara Bio Inc) protocol. For each sample, 1 µg of RNA was reverse-transcribed using the ImProm-II Reverse Transcription System (Promega).

The *pIgR*, *TLR2, TLR3, TLR4* and *TLR5* specific mRNA abundances were determined by real-time quantitative PCR (RT-qPCR), performed in a Light Cycler Real-Time PCR System (Roche). The reactions were carried out in duplicate in a 10 µL-volume containing about 100 ng of cDNA, 0.5 µM of each primer, and 5 µL of SYBR Premix Ex Taq II (Perfect Real Time, Takara Bio Inc). Reactions consisted of an initial denaturation step at 95 °C for 30 s and 40 cycles of 95 °C for 5 s and the annealing/extension temperature for 20 s. The primer sequences and annealing/extension temperatures are given in Table 1. Threshold cycles were converted to mRNA molecules/µL using a standard curve obtained as described previously [12]. Amplification specificity was checked by melting curve analysis at the end of the reaction for each primer set.

**Table 1:** Primers information and RT-qPCR conditions used in the trial.

Gene	NCBI accession number	Oligo sequence (5'→3')		Amplicon length	Annealing T
*TLR2*	NM_213761.1	Forward	GCGGAAGATAATGAACACCA	150 bp	62°C
		Reverse	AGAGCGATAAAGACCAGCAT		
*TLR3*	NM_001097444.1	Forward	CAAAACCAGCAACACGACTT	110 pb	59°C
		Reverse	GAAGCCAAGCAAAGGAATCA		
*TLR4*	NM_001113039.1	Forward	CTTCCTCCTGGTATCTGTGG	113 bp	60°C
		Reverse	CAAAGGCGTCATAGGTGCTT		
*TLR5*	NM_001123202.1	Forward	CAGCGACCAAAACAGATTGA	122 pb	59°C
		Reverse	TGCTCACCAGACAGACAACC		
*PIGR*	NM_214159.1	Forward	AGCCAACCTCACCAACTTCC	105 bp	62°C
		Reverse	CTGCTAATGCCCAGACCAC		
*HMBS2*	DQ845174	Forward	AGGATGGGCAACTCTACCTG	83 bp	62°C
		Reverse	GATGGTGGCCTGCATAGTCT		
*RPL4*	DQ845176	Forward	CAAGAGTAACTACAACCTTC	122 bp	60°C
		Reverse	GAACTCTACGATGAATCTTC		

**TLR2**, toll-like receptor 2; **TLR3**, toll-like receptor 3; **TLR4**, toll-like receptor 4; **TLR5**, toll-like receptor 5; PIGR, Polymeric immunoglobulin receptor; HMBS2, Hydroxymethylbilane synthase 2; RPL4, Ribosomal protein L4.

To ensure that the primers used for *pIgR* expression analysis were specific for the intended target, the products were sequenced (BMR Genomics) and confirmed to correspond with the expected porcine *pIgR* cDNA sequence. The expression data were normalized by geometric mean of the expression of the two housekeeping genes: *Hydroxymethylbilane Synthase* (*HMBS2*) and Ribosomal Protein L4 (RPL4). Primers and amplification conditions for the housekeeping genes are reported in Table 1.

### Immunohistochemistry

 For immunohistochemistry, the avidin-biotin-peroxidase complex (ABC) method was used as described elsewhere [13]. Briefly, 5 µm paraffin sections were dewaxed using xylene and rehydrated using descending alcohol-to-water gradient. Slides were heated in sodium citrate buffer (pH 6.0) for 10 min for antigen retrieval. Endogenous peroxidase was quenched by incubating the sections in 1% hydrogen peroxide in aqueous solution for 30 min at room temperature (RT). Nonspecific protein binding was blocked by treating the sections with 10% normal goat serum and 1% normal swine serum in PBS for 60 min. The sections were then incubated overnight at 4 °C with the polyclonal rabbit antiserum anti-pIgR (Sigma-Aldrich, HPA012012), diluted 1:600. After washing, the sections were incubated at room temperature for 1 h with a biotin-conjugated secondary antibody goat anti-rabbit IgG (Vector Laboratories) diluted 1:500 and then treated with ABC (Vector elite kit, Vector Laboratories) for 30 min at room temperature. The immune reactions were visualized applying a 3,3’-diaminobenzidine chromogen solution (Vector DAB kit, Vector Laboratories). Slides not treated with primary antibody served as negative control. The specific reactivity of the antibody for the sequence was assessed by inhibition test. Briefly, the primary antibody was incubated overnight at +4°C with an equimolar or excess of synthetic peptide (HPA012012, Atlas Antibodies), before proceeding with the immunochemistry reaction. No staining was observed for both negative and peptide-blocking reactions.

### In situ hybridization

A Locked Nucleic Acid (LNA)-enhanced DNA oligonucleotide probe, containing 30% of LNA nucleotide and digoxigenin-labelled at the 5’end was purchased from Exiqon Inc. 

The anti-sense probe (TTCTCTGGGAAGTTGGTGAGGT) was designed on the porcine *pIgR* mRNA reference sequences (GenBank: NM_214159; http://www.ncbi.nlm.nih.gov/) using the online probe designer tool on the Exiqon website (http://www.exiqon.com).

Paraffin sections were dewaxed, rehydrated and post-fixed in 4% paraformaldehyde in DEPC-treated PBS 10 min at RT. Sections were acetylated for 10 min in 0.1 M triethanolamine buffer at pH 8.0 containing 0.25% (v/v) acetic anhydride, incubated with proteinase K (5 µg/mL in PBS) for 6 min at 37 °C, and then in 4% paraformaldehyde for 5 min at RT. After washing with PBS, slides were incubated for 1 hour at 37 °C with the hybridization buffer containing 40% formamide, 5× saline sodium citrate (SSC), 2.5× Denhardt’s solution, 250 µg/mL yeast RNA, 500 µg/mL salmon sperm DNA and 1% blocking reagent (Roche). The sections were then incubated for 20 h at 55 °C in the hybridization buffer containing 80 nM of the denatured LNA probe and 0.1% Tween. The sections were soaked in 5× SSC at 60 °C, quickly washed in 1× SSC at RT, incubated 10 min in 1× SSC at 55 °C, 1 h in 0.2× SSC at 55 °C and washed in TBS buffer (Tris-HCl 0.1 M; NaCl 0.15 M). 

After incubation in blocking buffer (1% blocking reagent and 0.1% Tween in TBS) for 30 min, the sections were incubated overnight at 4 °C with sheep alkaline phosphatase-conjugated anti-digoxigenin polyclonal antibody (Roche, cat. 11093274910) diluted 1:1000 in blocking buffer. Sections were stained for 24 h at RT with the NBT/BCIP Ready-to-Use Tablets (Roche) following manufacturers’ instruction. Speciﬁcity of the probe was verified using the sense probe in a concentration gradient ranging from 500 pM to 100 nM. Negative controls were performed following the same procedure but omitting either the probe or the anti-digoxigenin antibody.

### Statistical analysis

Expression data were analyzed by analysis of variance using the GLM procedure of SAS (SAS Inst. Inc.) with a factorial design, including the antibiotic treatment of sows, the age of piglets, their interaction, and sows within treatment. Age was considered as a repeated measurement within each sow. Because the effect of the antibiotic supplementation to the sows and of its interaction with piglets age were not statistically significant, a second run of statistical analysis was done, excluding these factors. Thus, here we presented the pooled results on offspring from control sows and antibiotic-treated sows, except for TLR5 in Cd, where an interaction with the treatment of sows was seen, and only values obtained from control group were used. The data concerning antibiotic supplementation of the sows are beyond the goals of this paper. 

The following three orthogonal contrasts were tested: (1) linear and (2) quadratic effect among the three ages during suckling (14, 21 and 28 days); (3) “Weaned vs. Suckled”, between the post-weaning age (day 42) and the three ages during suckling.

Pearson's correlation coefficient *r* values between pIgR expression and TLRs expression was calculated by CORR procedure of SAS for each gastric site. *P* values ≤ 0.05 were considered statistically significant.

## Results

### Gene expression

**Figure 1:**
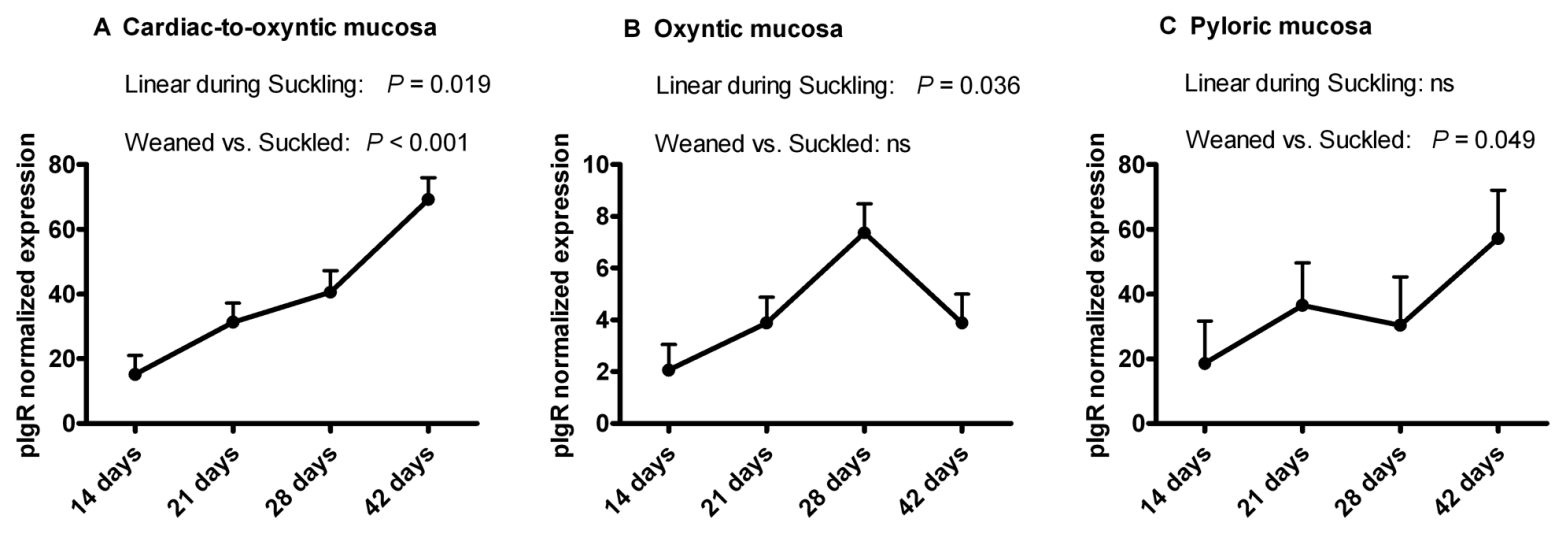
Polymeric immunoglobulin receptor (pIgR) mRNA expression in pig gastric mucosa. PIgR mRNA expression was determined in the mucosa at three gastric sites in piglets during the suckling period (14, 21 and 28 days of age) and after weaning (42 days of age). In cardiac-to-oxyntic mucosa, pIgR expression increased linearly during suckling (P=0.019) and also increased post-weaning (P=0.001). In pyloric mucosa, pIgR expression increased post-weaning (P=0.049). In oxyntic mucosa, pIgR expression increased linearly during suckling (P=0.036). For the three gastric sites, no significant quadratic effect during suckling was detected (*P*>0.05).

All the piglets remained healthy throughout the trial and were used for the expression analysis. The normalized *pIgR* mRNA expression values at four different ages and in three different gastric sites are reported in Figure 1. On average, mRNA expression in Ox was 10-fold lower than the expression in Cd and in Py. No significant quadratic effect among the three ages during suckling was detected in the three gastric sites (*P*>0.05).

In the Cd, the *pIgR* expression increased linearly during suckling (*P*=0.019) and it increased post-weaning compared with pre-weaning (*P*=0.001). Also in the Py, the *pIgR* expression increased at post-weaning age (*P*=0.049). In the Ox, the *pIgR* expression increased linearly during suckling (*P*=0.036), and it showed a marked decrease after weaning, contrary to the other two sites.

**Figure 2:**
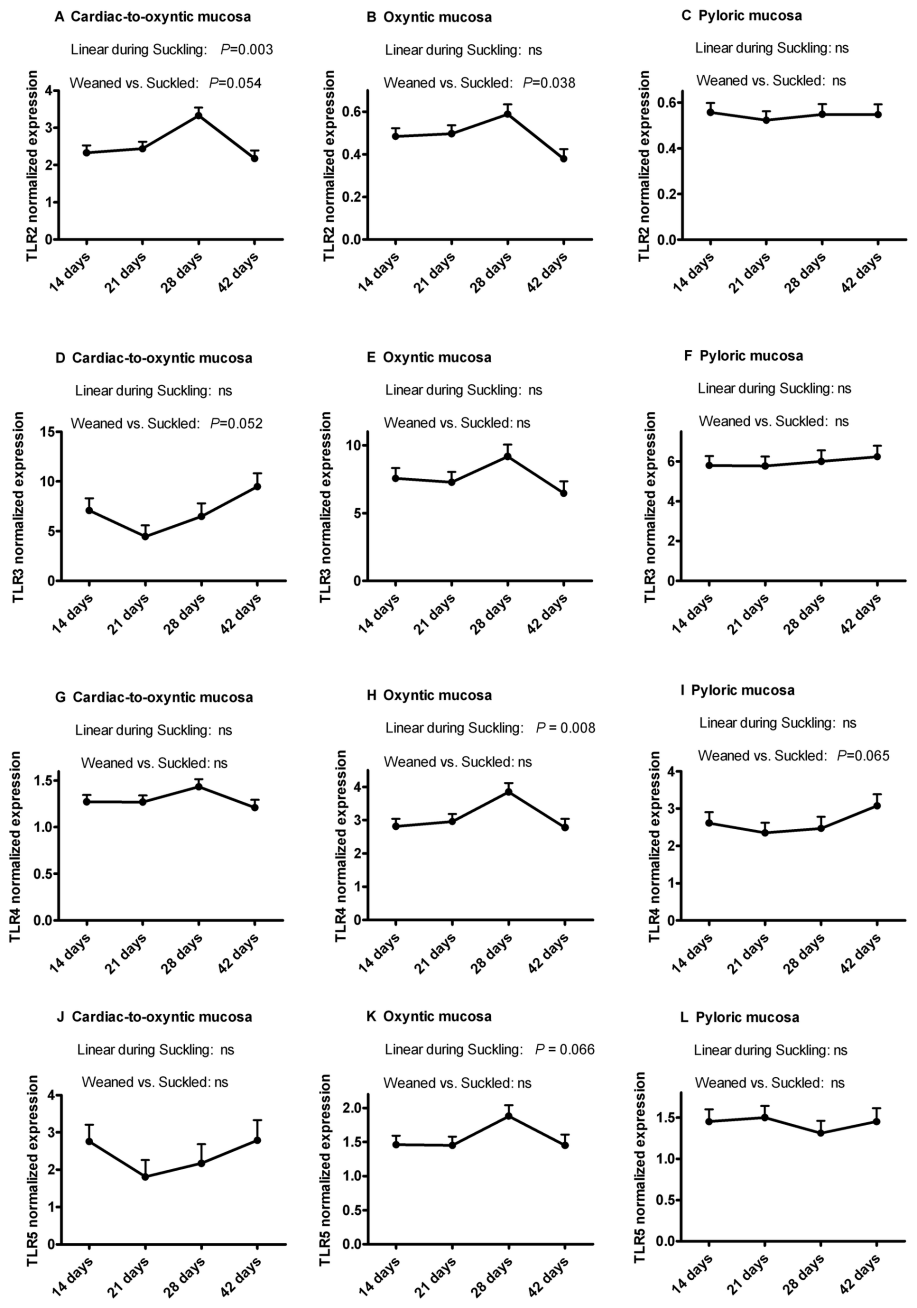
Toll-like Receptor (TLR) mRNA expression in pig gastric mucosa. Expression of *TLR2* (A-C), *TLR3* (D-F), *TLR4* (G-I) and *TLR5* (J-L) mRNA expression was determined in three sites of the gastric mucosa of piglets during the suckling period (14, 21 and 28 days of age) and after weaning (42 days of age). TLRs expression was modulated during development in the oxyntic mucosa, where *TLR2* decreased after weaning (P=0.038) and *TLR4* linearly increased during suckling (P=0.008), and in the cardiac-to-oxyntic mucosa, where *TLR2* linearly increased during suckling (P=0.003). For the three gastric sites, no significant quadratic effect during suckling was detected (*P*>0.05).

The normalized *TLR2*, *TLR3*, *TLR4* and *TLR5* mRNA expression values at four different ages and in three different gastric sites are reported in Figure 2. On average, mRNA expression of *TLR3* (compared with the other TLRs) was the highest for all the time points, while the lowest expression was observed for TLR2 in Py and Ox. In Py, the effect of age was not significant (trend for *TLR4*, *P*=0.065). In Cd, *TLR2* expression peaked at the end of the weaning period (*P*=0.003) and then decreased (*P*=0.054), while *TLR3* expression tended to increase from suckling to weaned pigs (*P*=0.053). For *TLR4* and *TLR5*, no age effect was found. In Ox, the highest values were observed at the end of the suckling period, with a linear increase during suckling (*TLR4*, *P*=0.008; trend for *TLR5*, *P*=0.066), or in comparison of weaned vs. suckled (*TLR2*, *P*=0.038).

Low but still significant correlations were seen between *pIgR* and *TLR2* (*r*=-0.24; *P*=0.02), *TLR4* (*r*=0.27; *P*=0.01) and *TLR5* (*r*=-0.28; *P*=0.01) expression values in Py (data not shown).

**Figure 3:**
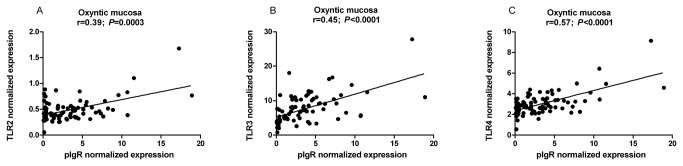
Pearson's correlation analysis between pIgR and TLR expressions in different gastric sites. Significant positive correlations with pIgR expression were found in the oxyntic region for *TLR 2* (A), *TLR3* (B), and *TLR4* (C).

Significant correlations were found between the expression level of *pIgR* and those of *TLR2* (*r*=0.39; *P*=0.0003), *TLR3* (*r*=0.45; *P*<0.0001) and *TLR4* (*r*=0.57; *P*<0.0001) in Ox, as shown in Figure 3 (A-C). 

### PIgR protein localization

**Figure 4:**
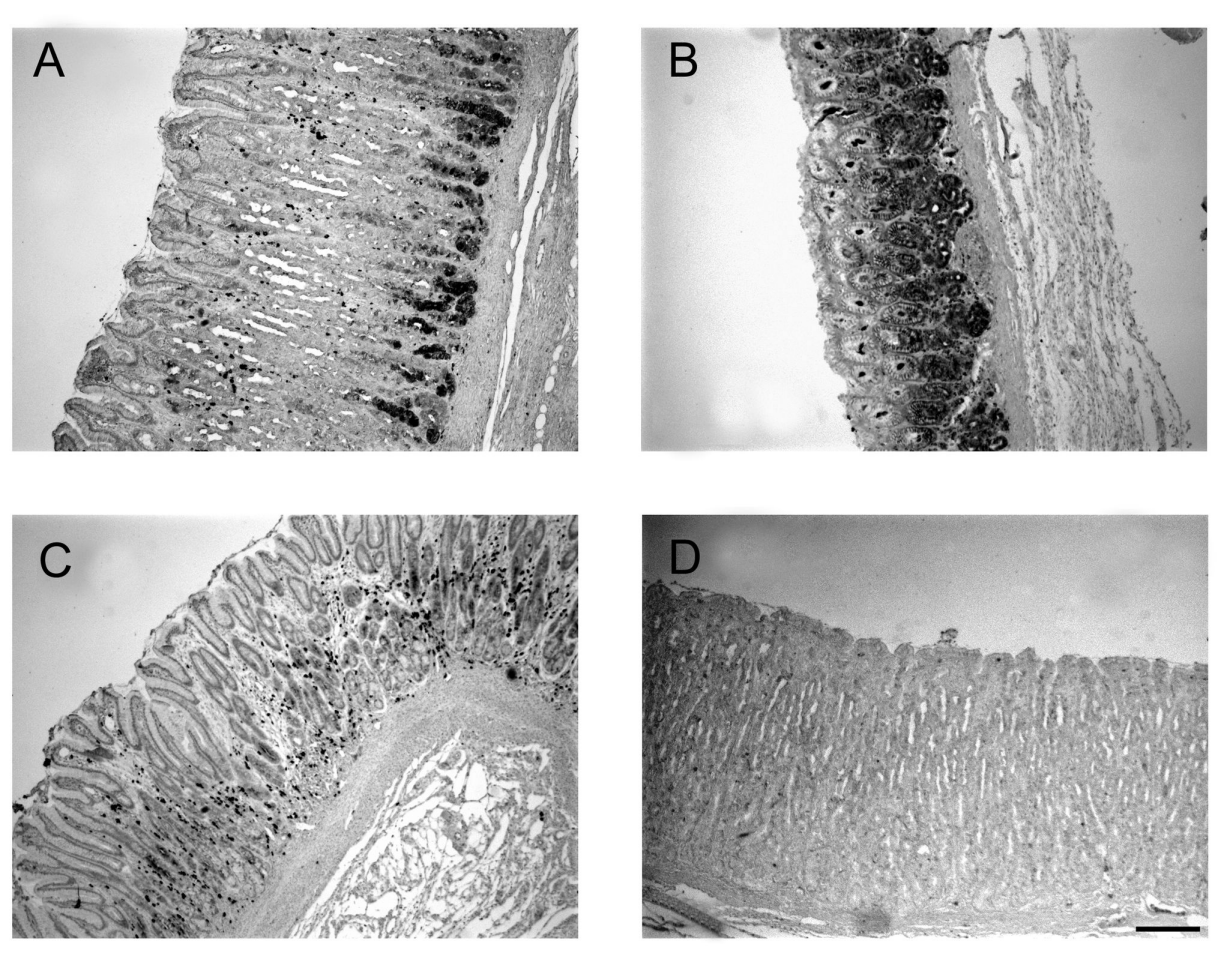
Localization of polymeric immunoglobulin receptor (pIgR) protein in three sites of the gastric mucosa of a representative pig aged 42 days. PIgR protein immunostaining in a 42 day-old representative piglet, in the transition from cardiac to oxyntic mucosa (A), in the proper glandular region (oxyntic mucosa) (B) and in the pyloric region (C). Negative control is with the omission of primary antibody (D). Bar indicates 200 µm (50× magnification).

Given that the gene expression studies of pIgR had shown the highest level of expression in 42 day-old piglets, these were selected for the immuno-histochemical localization of the pIgR protein in the three sites of the gastric mucosa (Figure 4). The analysis confirmed the presence of pIgR in the stomach. The pIgR protein was located in the gastric glands and the staining signal was stronger in the Cd compared with the other sites, in agreement with the RT-qPCR results. In the Cd region, pIgR was located in the tubular mucous-producing glands that were loosely packed within the lamina propria (Figure 4 A). In the Ox region, the protein was found at the bottom of the tightly packed glands (Figure 4 B). In the Py region, the staining was less defined and showed a spotted distribution pattern (Figure 4C). Additional immune-staining results are reported in Figure S1.

### In situ localization of pIgR mRNA

**Figure 5:**
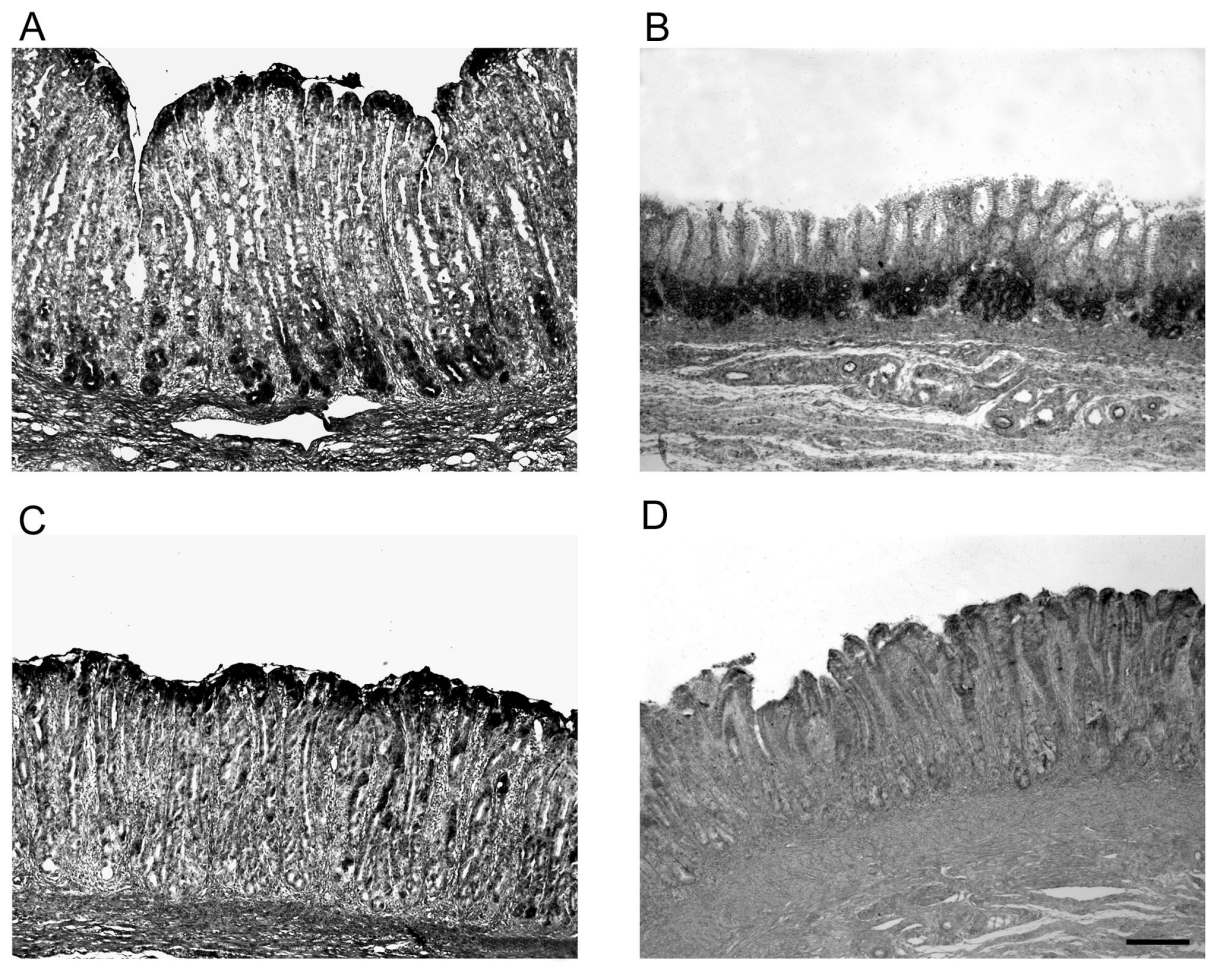
Localization of polymeric immunoglobulin receptor (pIgR) mRNA by *in situ hybridization* in the gastric mucosae of a representative pig aged 42 days. PIgR mRNA localization in a 42 day-old representative piglet, in the transition from cardiac to oxyntic mucosa (A), in the proper glandular region (oxyntic mucosa) (B) and in the pyloric region (C). Control with the sense probe is to ensure specificity of the reaction (D). Bar indicates 200 µm (50× magnification).

To determine the sites of active synthesis of the pIgR protein in situ hybridization for *pIgR* mRNA was performed on tissues from the three sites of the stomach from piglets aged 42 days, using serial sections from the samples used for the immunohistochemical protein localization. The mRNA was detected in the gastric glands within the lamina propria (Figure 5), mirroring pIgR protein localization (Figure 4) and confirming the active synthesis of this protein in these cells. Comparison between pIgR mRNA and protein localization in Cd at higher resolution is reported in Figure S2.

## Discussion

As a consequence of its inhospitable acidic environment the stomach has often been considered an “almost aseptic organ”, and little attention has been given to its potential role in immunity. To gain insight into the role of the stomach in the defense against pathogens in young piglets, we hypothesized that the gastric mucosa is provided with the basic machinery of the innate and adaptive mucosal immune system. This hypothesis was supported by a previous report that described the distribution of the gastric-associated lymphoid tissue in conventional piglets [10]. For this study we focused on pIgR as a critical marker of the mucosal immune system, determining the time course of it’s appearance and expression pattern in the gastric mucosa.

In this study, *pIgR* mRNA was detected in the stomach of piglets for the first time, in good agreement with data in mice [9]. Here, pIgR transcription levels were found to be age-dependent along the maturation of gastric mucosa in growing piglets. This suggests that the progressive functional maturation of mucosa-associated lymphoid tissue is linked to an increase in pIgR gene expression.

After birth, the gastrointestinal tract undergoes a complex process of growth and differentiation and several genes involved in this process are developmentally regulated, mainly at the level of transcription [14]. This development process includes the maturation of the mucosal adaptive immune system, which is immature in neonatal and suckling animals [15]. In the intestine, the organized lymphoid tissue expands and antigen-presenting cells and T-cells begin to infiltrate the mucosa within days after birth [16], a maturation process that is dependent on colonization by microorganisms [13]. The maturation of the mucosa from birth to post-weaning age is reflected in an increased expression of *pIgR* mRNA as reported here. Similarly in mice the *pIgR* expression was found to rise from the pre- to the post-weaning period in the small intestine [14], while in rat intestine the *pIgR* is expressed only after weaning [17].

The pIgR promotes the intracellular neutralization and transcellular excretion of antigens and pathogens and ensures continuous delivery of sIgA to the luminal epithelial surface [6]. On the other hand, free SC produced by transcytosis of pIgR in the absence of sIgA ligand interacts directly with several bacteria or their products, thus being an important component of innate anti-microbial defense [18]. Therefore, the up-regulation of pIgR expression could increase both adaptive and innate mucosal immune responses.

We observed regional differences in the pIgR expression profiles among the functionally different parts of the stomach. The oxyntic mucosa (Ox), pyloric mucosa (Py) and cardiac-to-oxyntic transition mucosa (Cd) corresponded to the sites where a previous study found several submucosal lymphoid follicles, defined as sites 1, 4-5 and 7, respectively [10]. The *pIgR* gene expression was highest in the pyloric mucosa and in the cardiac-and-oxyntic mucosa transition than in the oxyntic mucosa. It also increased from the pre- to the post-weaning period, probably mirroring the functional development of mucosal immune system as well as the adaptation to the diet. On the other hand, the reduced *pIgR* mRNA expression in the oxyntic mucosa of post-weaning pigs may be related to the rise in hydrochloride acid (HCl) secretion that occurs at the end of the suckling period in this region. Increased HCl production could lead to the reduction of bacteria-driven local Ig secretion, mirroring the decreased *pIgR* expression reported here.

The ability of *pIgR* gene to react to the presence of pathogens in the stomach (typically *Helicobacter* spp.) has been already described in mice [19]. Gastritis can also stimulate immunological activity in the human antrum, where abundant IgA-positive plasma cells were detected and pIgA was secreted by pIgR-expressing epithelium [20]. In contrast, the impact of commensal bacteria on the development of immunity in the stomach has received little attention, but it is perhaps not surprising to find significant pIgR activity in pigs that are kept in a “dirty” environment where the bacterial load is likely to be high. Our data show that the stomach of healthy pigs is able to react to the increasing contact with commensal bacteria and, presumably, with the increasing complexity of microbiota observed in the suckling and post-weaning periods.

Bacterial components and/or double-stranded RNA (sign of viral presence) may provide signals through TLRs such as TLR3 and TLR4, leading to the induction of *pIgR* gene transcription, involving nuclear factor-κB [21,22]. This implies a cooperation of signaling from microbial products and host cytokines in *pIgR* gene regulation [21], and in cell culture the activation of TLRs resulted in stimulation of pIgR transcytosis [23]. Here we showed that the *TLR2* to *TLR5* genes were almost all already activated at transcriptional level at the earliest ages studied. Thus, they are not likely to limit the activation of pIgR in the different gastric compartments from 2 weeks of age onward. However, depending on the mucosal site some age effects were observed for each TLR, in particular for *TLR2* in the cardiac-to-oxyntic transition region and for *TLR2* and *4* in the oxyntic region. The present study confirmed preliminary data previously reported regarding TLRs expression in the stomach [24,25] and extended them to three different gastric locations using greater numbers of individuals. Moreover, age effects on TLRs expression have been investigated in pigs, but over a different time scale (at 1 day, 2 months and 5 months of age [24]; or pre-weaning [25]), and only *TLR3* gene expression was found greater in adult pigs compared to younger pigs [24]. Toll-like receptor-3 (TLR3), which is activated by double-stranded RNA and plays a role in anti-viral immunity, was reported to be expressed to greater levels in the porcine stomach than in the intestine [24,26]. This confirmed the importance of the stomach as a first site where viruses can be detected and the innate immune system activated. Moreover, in this study, the expression of TLR3 in the stomach was higher compared to the other TLRs, in agreement with recent data [24], but no differences were seen between the different gastric sites and ages. This suggests that even by 14 days of age the gastric mucosa is able to synthesize *TLR3* mRNA as efficiently as in adult pigs, possibly due to a high exposure to environmental viruses. The pattern of induction of TLR3 with age did not fully resemble that of pIgR and, therefore, we can conclude that pIgR induction during development is only partially affected by TLR3. It is also noteworthy that in the oxyntic mucosa, the pattern of TLRs expression at different ages resembled that of pIgR, especially for TLR2, 3 and 4, with values that tended to peak at 28 days of age and then decreasing post-weaning. This was confirmed by significant and positive correlations between pIgR and TLRs gene expressions. This supports the suggestion that increased oxyntic mucosa HCl secretion induced by solid feed intake would reduce the local immune stimulation generated by microorganisms.

The *TLR5* that recognizes flagellin of flagellated bacteria was found to be more expressed in the stomach than in the intestine of mice [27]. Overall, the modest variations in gene expression of the TLRs we determined here may indicate that these TLRs are back-regulated to maintain a baseline for their activation. In fact several mechanisms to induce bacterial tolerance have been evidenced for TLRs in the intestine [28]. This may also explain, at least in part the weak or non-significant correlations observed between gene expressions for TLRs and pIgR in pyloric and cardiac-to-oxyntic transition mucosae. But several other factors may also regulate pIgR: interferon-γ, steroid hormones, pro-inflammatory cytokines, interleukin 17 [29].

This is the first report on pIgR localization in the gastric mucosa. The expression of pIgR at the various gastric sites was confirmed by protein immunostaining in 42 days-old pigs where it was mainly found within the gastric glands in the lamina propria. The expression of pIgR has been reported in the epithelial and glandular cells in various organs, including kidney, lung, pancreas, and rat but not human liver [4,9], and especially in the columnar epithelial cells of the intestine [5,6,30]. Gastric pIgR is likely involved in the release of sIgs, as well as of free form of the SC, within the coiled gastric glands that empty into the base of the so-called foveolae, gastric pits representing invaginations of the surface epithelium. The active synthesis of pIgR protein in the glandular cells of gastric mucosa was confirmed in the present study by the *in situ* localization of the *pIgR* gene transcript within the same cells.

In conclusion, we have demonstrated for the first time that pIgR is expressed in the stomach of the piglet at different ages. The presence of pIgR was confirmed by mRNA and protein localization, with a stronger protein staining in the cardiac-oxyntic transition mucosa, previously found to be rich in submucosal lymphoid follicles. The development of detection capacity for microorganisms in the different gastric mucosae by TLRs may at least in part explain variation in pIgR expression. Collectively, our data suggest different mechanisms of immune responses to antigens in the different gastric sites. They also strengthen the idea that the stomach may play a primary role in the adaptive and innate mucosal immunity in young growing pigs.

## Supporting Information

Figure S1 **Localization of polymeric immunoglobulin receptor (pIgR) protein in three sites of the gastric mucosa of a 42 day-old representative piglet.** PIgR protein immunostaining in the transition from cardiac to oxyntic mucosa (A), in the proper glandular region (B) and in the pyloric region (C). Bar indicates 100 μm (100× magnification).
(TIF)

Figure S2 **Localization of polymeric immunoglobulin receptor (pIgR) mRNA by *insituhybridization* (**A**) and of pIgR protein by immunohistochemistry (**B**) in serial sections of the cardiac-to-oxyntic transition mucosa of a 42 day-old representative piglet.** Bar indicates 100 μm (100× magnification).
(TIF)

## References

[1] Hooper LV, Gordon JI (2001) Commensal host-bacterial relationships in the gut. Science 292: 1115-1118. doi:10.1126/science.1058709. PubMed: 11352068. 11352068

[2] Bailey M, Haverson K, Inman C, Harris C, Jones P et al. (2005) The development of the mucosal immune system pre- and post-weaning: balancing regulatory and effector function. Proc Nutr Soc 64: 451-457. doi:10.1079/PNS2005452. PubMed: 16313686. 16313686

[3] Burkey TE, Skjolaas KA, Minton JE (2009) Board-invited review: porcine mucosal immunity of the gastrointestinal tract. J Anim Sci 87: 1493-1501. doi:10.2527/jas.2008-1330. PubMed: 19028849. 19028849

[4] Strugnell RA, Wijburg OL (2010) The role of secretory antibodies in infection immunity. Nat Rev Microbiol 8: 656-667. doi:10.1038/nrmicro2384. PubMed: 20694027. 20694027

[5] Asano M, Komiyama K (2011) Polymeric immunoglobulin receptor. J Oral Sci 53: 147-156. doi:10.2334/josnusd.53.147. PubMed: 21712618. 21712618

[6] Kaetzel CS (2005) The polymeric immunoglobulin receptor: bridging innate and adaptive immune responses at mucosal surfaces. Immunol Rev 206: 83-99. doi:10.1111/j.0105-2896.2005.00278.x. PubMed: 16048543. 16048543

[7] Karlsson MR, Johansen FE, Kahu H, Macpherson A, Brandtzaeg P (2010) Hypersensitivity and oral tolerance in the absence of a secretory immune system. Allergy 65: 561-570. doi:10.1111/j.1398-9995.2009.02225.x. PubMed: 19886928. 19886928

[8] Krajci P, Solberg R, Sandberg M, Oyen O, Jahnsen T (1989) Molecular cloning of the human transmembrane secretory component (poly-Ig receptor) and its mRNA expression in human tissues 158 Biochem Bioph Res Co. pp. 783-789. PubMed: 2920039. 10.1016/0006-291x(89)92790-3 2920039

[9] Asano M, Saito M, Suguro H, Nomura H, Inage T et al. (2004) Active synthesis of mouse polymeric immunoglobulin receptor in the epithelial cells of the distal urinary tubule in kidney. Scand J Immunol 60: 267-272. doi:10.1111/j.0300-9475.2004.01456.x. PubMed: 15320883. 15320883

[10] Mazzoni M, Bosi P, De Sordi N, Lalatta-Costerbosa G (2011) Distribution, organization and innervation of gastric MALT in conventional piglet. J Anat 219: 611-621. doi:10.1111/j.1469-7580.2011.01415.x. PubMed: 21781093. 21781093 PMC3222840

[11] Bruno MEC, Frantz AL, Rogier EW, Johansen FE, Kaetzel CS (2011) Regulation of the polymeric immunoglobulin receptor by the classical and alternative NF-κB pathways in intestinal epithelial cells. Mucosal Immunol 4: 468-478. doi:10.1038/mi.2011.8. PubMed: 21451502. 21451502 PMC3125104

[12] Trevisi P, De Filippi S, Minieri L, Mazzoni M, Modesto M (2008) Effect of fructo-oligosaccharides and different doses of Bifidobacterium animalis in a weaning diet on bacterial translocation and Toll-like receptor gene expression in pigs. Nutrition 24: 1023-1029. doi:10.1016/j.nut.2008.04.008. PubMed: 18562167. 18562167

[13] Bosi P, Mazzoni M, De Filippi S, Trevisi P, Casini L et al. (2006) A continuous dietary supply of free calcium formate negatively affects the parietal cell population and gastric RNA expression for H^+^/K^+^-ATPase in weaning pigs. J Nutr 136: 1229-1235. PubMed: 16614409. 16614409 10.1093/jn/136.5.1229

[14] Jenkins SL, Wang J, Vazir M, Vela J, Sahagun O et al. (2003) Role of passive and adaptive immunity in influencing enterocyte-specific gene expression. Am J Physiol Gastrointest Liver Physiol 285: G714-G725. PubMed: 12969828. 12969828 10.1152/ajpgi.00130.2003

[15] Vega-López MA, Bailey M, Telemo E, Stokes CR (1995) Effect of early weaning on the development of immune cells in the pig small intestine. Vet Immunol Immunopathol 44: 319-327. doi:10.1016/0165-2427(94)05309-G. PubMed: 7747409. 7747409

[16] Rothkötter HJ, Ulbrich H, Pabst R (1991) The postnatal development of gut lamina propria lymphocytes: number, proliferation, and T and B cell subsets in conventional and germ-free pigs. Pediatr Res 29: 237-242. doi:10.1203/00006450-199103000-00004. PubMed: 2034471. 2034471

[17] Huling S, Fournier GR, Feren A, Chuntharapai A, Jones AL (1992) Ontogeny of the secretory immune system: maturation of a functional polymeric immunoglobulin receptor regulated by gene expression. Proc Natl Acad Sci U_S_A 89: 4260-4264. doi:10.1073/pnas.89.10.4260. PubMed: 1374892. 1374892 PMC49061

[18] Phalipon A, Corthésy B (2003) Novel functions of the polymeric Ig receptor: well beyond transport of immunoglobulins. Trends Immunol 24: 55-58. doi:10.1016/S1471-4906(02)00031-5. PubMed: 12547499. 12547499

[19] Björkholm BM, Guruge JL, Oh JD, Syder AJ, Salama N et al. (2002) Colonization of germ-free transgenic mice with genotyped Helicobacter pylori strains from a case-control study of gastric cancer reveals a correlation between host responses and HsdS components of type I restriction-modification systems. J Biol Chem 277: 34191-34197. doi:10.1074/jbc.M203613200. PubMed: 12105196. 12105196

[20] Valnes K, Brandtzaeg P, Elgjo K, Stave R (1984) Specific and nonspecific humoral defense factors in the epithelium of normal and inflamed gastric mucosa. Immunohistochemical localization of immunoglobulins, secretory component, lysozyme, and lactoferrin. Gastroenterology 86: 402-412. PubMed: 6198236. 6198236

[21] Schneeman TA, Bruno ME, Schjerven H, Johansen FE, Chady L et al. (2005) Regulation of the polymeric Ig receptor by signaling through Toll-like receptors 3 and 4: linking innate and adaptive immune responses. J Immunol 175: 376–384. PubMed: 15972671. 15972671 10.4049/jimmunol.175.1.376

[22] Bruno ME, Rogier EW, Frantz AL, Stefka AT, Thompson SN et al. (2010) Regulation of the polymeric immunoglobulin receptor in intestinal epithelial cells by Enterobacteriaceae: implications for mucosal homeostasis. Immunol Investig 39: 356-382. doi:10.3109/08820131003622809. PubMed: 20450283. 20450283

[23] Diebel LN, Liberati DM (2011) Disparate effects of bacteria and toll-like receptor-dependant bacterial ligand stimulation on immunoglobulin a transcytosis. J Trauma 70: 691-700. doi:10.1097/TA.0b013e31820c780e. PubMed: 21610360. 21610360

[24] Uddin MJ, Kaewmala K, Tesfaye D, Tholen E, Looft C et al. (2013) Expression patterns of porcine Toll-like receptors family set of genes (TLR1-10) in gut-associated lymphoid tissues alter with age. Res Vet Sci 95: 92-102. doi:10.1016/j.rvsc.2013.01.027. PubMed: 23433683. 23433683

[25] Pan ZY, Chen Z, Liu L, Cao YZ, Xie KZ et al. (2011) Differentiation of porcine TLR4 gene expression in piglets of different ages. J Anim Vet Adv 10: 2312-2316. doi:10.3923/javaa.2011.2312.2316.

[26] Sang Y, Yang J, Ross CR, Rowland RR, Blecha F (2008) Molecular identification and functional expression of porcine Toll-like receptor (TLR) 3 and TLR7. Vet Immunol Immunopathol 125: 162–167. doi:10.1016/j.vetimm.2008.04.017. PubMed: 18533275. 18533275

[27] Goebel M, Stengel A, Lambrecht NW, Sachs G (2011) Selective gene expression by rat gastric corpus epithelium. Physiol Genomics 43: 237-254. doi:10.1152/physiolgenomics.00193.2010. PubMed: 21177383. 21177383 PMC3068518

[28] Miron N, Cristea V (2012) Enterocytes: active cells in tolerance to food and microbial antigens in the gut. Clin Exp Immunol 167: 405-412. doi:10.1111/j.1365-2249.2011.04523.x. PubMed: 22288583. 22288583 PMC3374272

[29] Brandtzaeg P (2013) Secretory IgA: designed for anti-microbial defense. Front Immunol 4: 222 PubMed: 23964273. 23964273 10.3389/fimmu.2013.00222 PMC3734371

[30] Reséndiz-Albor AA, Reina-Garfias H, Rojas-Hernández S, Jarillo-Luna A, Rivera-Aguilar V (2010) Regionalization of pIgR expression in the mucosa of mouse small intestine. Immunol Lett 128: 59-67. doi:10.1016/j.imlet.2009.11.005. PubMed: 19925828. 19925828

