# Supplementary figures and images for: Age-Related Expression of the Polymeric Immunoglobulin Receptor (pIgR) in the Gastric Mucosa of Young Pigs
Source: PLoS One. 2013 Nov 13;8(11):e81473. doi: 10.1371/journal.pone.0081473 (PMC3827463; doi:10.1371/journal.pone.0081473)

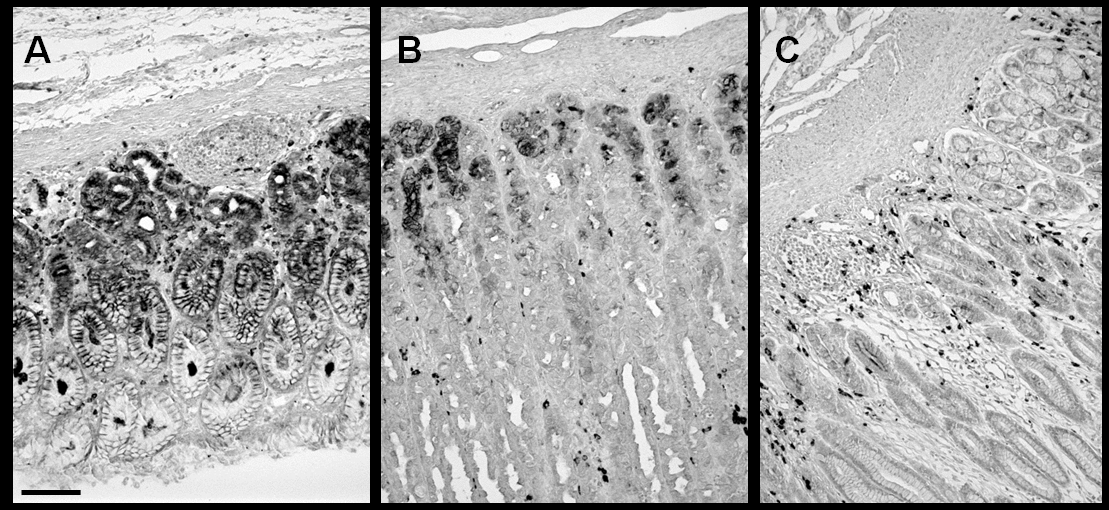

Supplement: Figure S1 — Localization of polymeric immunoglobulin receptor (pIgR) protein in three sites of the gastric mucosa of a 42 day-old representative piglet. PIgR protein immunostaining in the transition from cardiac to oxyntic mucosa (A), in the proper glandular region (B) and in the pyloric region (C). Bar indicates 100 μm (100× magnification). (TIF) [file pone.0081473.s001.tif]

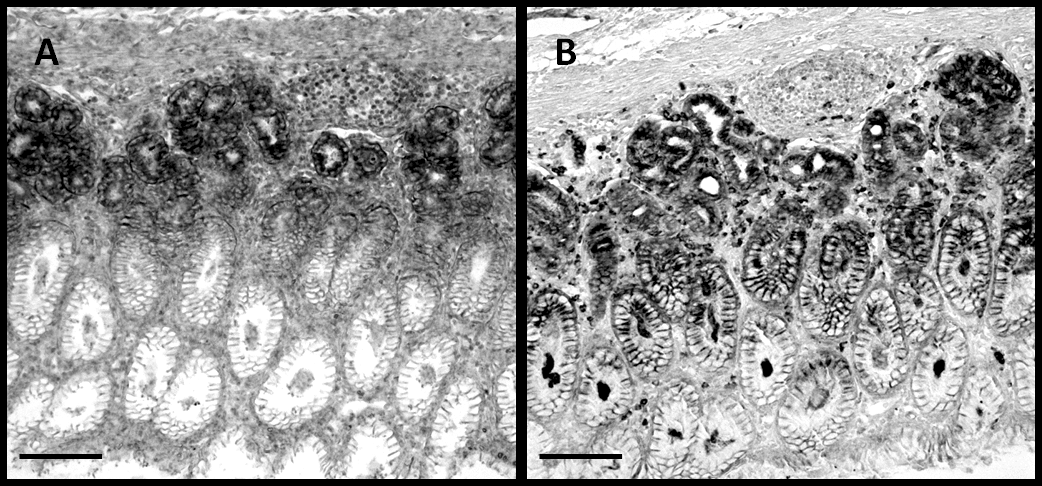

Supplement: Figure S2 — Localization of polymeric immunoglobulin receptor (pIgR) mRNA by insituhybridization (A) and of pIgR protein by immunohistochemistry (B) in serial sections of the cardiac-to-oxyntic transition mucosa of a 42 day-old representative piglet. Bar indicates 100 μm (100× magnification). (TIF) [file pone.0081473.s002.tif]
